# Factors Associated With Receipt of Adjuvant Radiation in Locally Advanced Rectal Cancer

**DOI:** 10.1002/jso.70365

**Published:** 2026-09-06

**Authors:** Maryam Sherwani, Danish Ali, Hanjoo Lee, Alexander T. Hawkins, Aimal Khan

**Affiliations:** ^1^ Section of Surgical Sciences, Vanderbilt University Medical Center Nashville Tennessee USA; ^2^ University of Texas Health Science Center at San Antoni San Antonio Texas USA; ^3^ Department of Surgery Division of Colon & Rectal Surgery, Harbor‐UCLA Medical Center Torrance California USA

**Keywords:** adjuvant radiation, locally advanced rectal cancer, postoperative radiation, rectal cancer

## Abstract

**Background:**

Neoadjuvant radiotherapy (RT) has been the standard of care for locally advanced rectal cancer (LARC) for over two decades. Adjuvant RT use in LARC represents a deviation from standard care and warrants further investigation.

**Objectives:**

To identify factors associated with adjuvant RT utilization. We hypothesized that sociodemographic determinants contribute to differences in the sequence of radiation treatment among patients with rectal cancer.

**Methods:**

This retrospective cohort study utilized the National Cancer Database (2015–2022) to identify adults with clinical stage II–III rectal adenocarcinoma undergoing surgery and RT. Patients were categorized by RT sequence (neoadjuvant vs. adjuvant). Multivariable logistic regression identified factors associated with use of adjuvant RT.

**Results:**

Of the included 48 452 patients, 6.9% received adjuvant RT. Compared with the neoadjuvant group, adjuvant RT recipients exhibited higher rates of pathologic upstaging (20.5% vs. 10.3%), positive margins (14.7% vs. 6.3%), and lymphovascular invasion (17.4% vs. 11.3%; all *p* < 0.001). On multivariable analysis, positive margins (aOR 1.78, 95% CI 1.43–2.22), lymphovascular invasion (aOR 1.96, 95% CI 1.66–2.31), upstaging (aOR 1.63, 1.31–2.02) were independently associated with adjuvant RT.

**Conclusions:**

Adjuvant RT appeared to be used in patients who were found to have high‐risk tumor pathology postoperatively.

**Abbreviations**
CoC: Commission on Cancer
LARC: locally advanced rectal cancer
NCDB: National Cancer Database
RT: radiotherapy
STROBE: Strengthening the Reporting of Observational Studies in Epidemiology

## 1 Introduction

Radiotherapy (RT) has long been a cornerstone of rectal cancer management, with its role continually reshaped by trials and recommendations. Introduced in the 1980s as an adjunct to surgery, RT reduced local recurrence rates that had previously exceeded 50% with surgery alone [1]. In 1990, the National Institutes of Health endorsed adjuvant chemoradiation as the standard treatment for patients with locally advanced rectal cancer (LARC; TNM stage II–III) [2]. The paradigm shifted just a few years later when the landmark Swedish Rectal Cancer Trial demonstrated that neoadjuvant RT significantly reduced recurrence and improved survival compared to surgery alone [3]. Building on these findings, the German CAO/ARO/AIO‐94 trial (2004) established that neoadjuvant chemoradiation decreased toxicity and improved local control and sphincter preservation compared with adjuvant therapy, leading to its adoption as the standard of care for LARC [4].

Today, the use of adjuvant chemoradiation has been primarily reported in cases of incidental nodal involvement or pathologic upstaging following surgery for early‐stage disease (TNM stage 0–I) [5, 6]. Its use is limited by notable drawbacks including increased overall toxicity; radiation injury to new colon anastomosis; potentially decreased efficacy due to a more radioresistant, hypoxic postsurgical bed; and perineal wound complications [7]. The omission of neoadjuvant RT in LARC, with subsequent RT administration in the adjuvant setting, represents a deviation from standard practice. Nevertheless, adjuvant RT continues to be administered in select patients and gaps remain in the literature regarding the factors influencing its use and the characteristics of patients receiving it.

This study evaluated factors associated with the use of adjuvant RT in the treatment of LARC in contemporary practice. Second, we sought to compare the clinical and pathological characteristics of patients who received neoadjuvant RT versus those who received adjuvant RT. We hypothesized that sociodemographic determinants contribute to disparities in care among patients with rectal cancer, as observed in other malignancies [8].

## 2 Materials and Methods

### 2.1 Study Design and Data Source

This was a retrospective cohort study conducted from the National Cancer Database (NCDB) using data from 2015 to 2022. The NCDB is a joint project of the Commission on Cancer (CoC), the American College of Surgeons, and the American Cancer Society [9]. The NCDB gathers and provides comprehensive, de‐identified information on patient demographics, tumor stage, and histopathology, treatment modalities, and patient outcomes for over 70% of cancer cases diagnosed annually in the United States (from participating hospitals) in a Health Insurance Portability and Accountability Act compliant format [10]. For this study, we retrieved data from the rectal cancer participant user data file.

Due to the retrospective deidentified nature of the databases, Institutional Review Board approval and patient informed consent were not required. This study adhered to the Strengthening the Reporting of Observational Studies in Epidemiology (STROBE) reporting guideline (Supporting Information S1) [11].

### 2.2 Study Population

We identified adult patients with primary rectal adenocarcinoma diagnosed between 2015 and 2022 in the NCDB. Patients were included if they had LARC (clinical stage II–III) and underwent surgery with either neoadjuvant or adjuvant RT. Exclusion criteria included stage 0, I, or IV disease; non‐adenocarcinoma histology; missing data on clinical stage; absence of surgery or RT; intraoperative RT; receipt of both neoadjuvant and adjuvant RT; or unknown sequence of surgery and RT. The final cohort was stratified based on the sequence of RT, neoadjuvant RT versus adjuvant RT (Figure 1).

**Figure 1:**
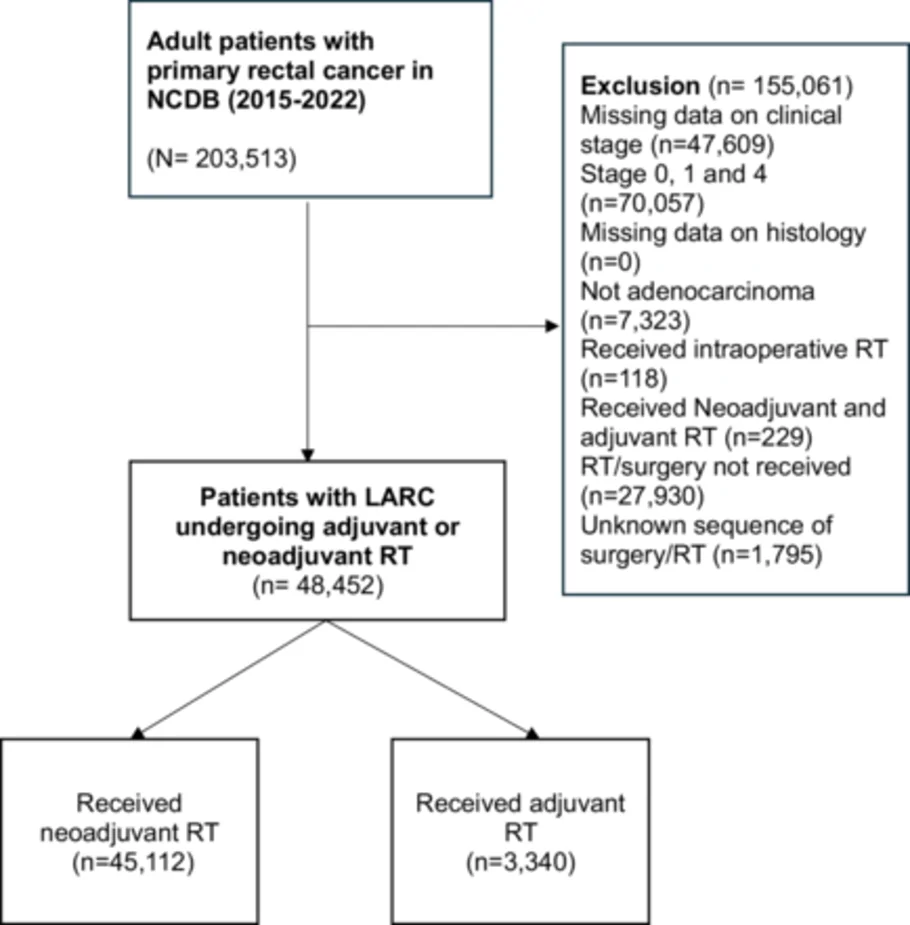
Study sample selection flowchart. LARC, locally advanced rectal cancer; NCDB, National Cancer Database; RT radiotherapy.

### 2.3 Study Outcomes

The primary outcome of this study was the identification of factors associated with adjuvant RT use among patients with LARC during the study period. Secondary outcomes included assessing the trends in utilization of adjuvant RT over the study period.

### 2.4 Variables of Interest

Regarding participant characteristics, the variables of interest included age at diagnosis (years), sex, race (White, Black and Others [a combination of all other categories in the database]), insurance status (uninsured, private, and governmental), Charlson‐Deyo score (0, 1, 2, and ≥ 3), median income (using the 2020 data; < $46 277, $46 277–$57 856, $57 857–$74 062, and ≥ $74 063). City or facility characteristics included population density (using the typology published by the USDA Economic Research Service in 2013: metro, urban, and rural), the percentage of adults in a zip code (25 years or older) who did not graduate high school (< 5.0%, 5.0%–9.0%, 9.1%–15.2%, and > 15.3%), distance from residence to hospital (miles) and facility type (community program, comprehensive program, academic/research program, and integrated network program; as deemed by the CoC). Tumor characteristics included clinical stage (according to the American Joint Committee on Cancer staging manual 8th Edition), pathologic upstaging (defined as pathologic stage higher than clinical stage), tumor histology (adenocarcinoma, mucinous, and signet‐cell adenocarcinomas), perineural invasion, lymphovascular invasion [12].

### 2.5 Statistical Analysis

Categorical data were presented as frequencies and compared using the Chi‐square test. Normality of continuous variables was assessed using the skewness–kurtosis test and visual inspection of histograms. As all continuous variables were non‐normally distributed, they were summarized as median (interquartile range, IQR) and compared using the Wilcoxon rank‐sum test. Multivariable logistic regression analysis was performed to identify factors associated with receipt of adjuvant RT and a forest plot was generated to illustrate its results. As sensitivity analysis, for variables with > 10% missingness, we employed multiple imputations by chained equations (MICE) to estimate missing values with probability estimates for regression analysis (Appendix A). All analyses were performed using Stata, release 14, statistical Software (StataCorp LLC, College Station, TX), and a *p* < 0.05 was considered significant throughout.

## 3 Results

A total of 203 513 adult patients with primary rectal cancer were identified in the NCDB between 2015 and 2022. After applying the inclusion and exclusion criteria, 48 452 patients were included in the analysis, with 45 112 (93.1%) receiving neoadjuvant and 3340 (6.9%) receiving adjuvant RT. Table 1 provides a detailed comparison of patient, city, and facility characteristics between the two groups. Compared to the neoadjuvant RT group, adjuvant RT recipients had higher rates of pathologic upstaging (20.5% vs. 10.3%, *p* < 0.001), positive surgical margins (14.7% vs. 6.3%, *p* < 0.001), and lymphovascular invasion (17.4% vs. 11.3%, *p* < 0.001). Significant but modest differences were noted in participant (age, race, type of insurance, income, and education), city/facility (population density, distance from hospital), and tumor (stage, histology) characteristics.

**Table 1:** Comparison of baseline patient, city and facility characteristics between the study cohorts.

Variable	Neoadjuvant RT (*n* = 45 112)	Adjuvant RT (*n* = 3340)	*p*‐value	Missing (%)
Participant characteristics				
Age, median (IQR), years	60 (17)	61 (17)	**< 0.001**	
Sex, *n* (%)				
Female	17 173 (38.1)	1300 (38.9)	0.326	
Race, *n* (%)				
White	38 074 (84.4)	2791 (83.6)	**0.010**	
Black	3501 (7.8)	306 (9.2)		
Others	3537 (7.8)	243 (7.3)		
Insurance, *n* (%)				
No insurance	1476 (3.3)	83 (2.5)	**< 0.001**	
Private	22 616 (50.1)	1590 (47.6)		
Government	20 605 (45.7)	1625 (48.7)		
Unknown	415 (0.9)	42 (1.3)		1.0
Charlson‐Deyo Score, *n* (%)				
0	35 461 (78.6)	2593 (77.6)	0.468	
1	6629 (14.7)	513 (15.4)		
2	1793 (4.0)	132 (4.0)		
3 or more	1229 (2.7)	102 (3.1)		
Median income^a^, *n* (%)				
< $46 277	6485 (14.4)	543 (16.3)	**0.004**	
$46 277–$57 856	8704 (19.3)	611 (18.3)		
$57 857–$74 062	9384 (20.8)	717 (21.5)		
$74 063+	13 484 (29.9)	938 (28.1)		
Unknown	7343 (15.6)	531 (15.9)		15.7
Population density, *n* (%)				
Metro	34 460 (76.4)	2614 (78.3)	**0.050**	
Urban	7813 (17.3)	526 (15.7)		
Rural	1169 (2.6)	83 (2.5)		
Unknown	1670 (3.7)	117 (3.5)		3.7
Percent with no high school degree^a^, *n* (%)				
> 15.3%	8269 (18.3)	666 (19.9)	**0.037**	
9.1%–15.2%	10 949 (24.3)	819 (24.5)		
5.0%–9.0%	11 132 (24.7)	803 (24.0)		
< 5%	7831 (17.4)	533 (16.0)		
Unknown	6931 (15.4)	519 (15.5)		15.4
Distance to hospital, median (IQR), miles	13.0 (25.6)	12.0 (21.5)	**< 0.001**	14.7
Type of facility, *n* (%)				
Community cancer program	2704 (6.0)	221 (6.6)	0.572	
Comprehensive community cancer program	16 038 (35.6)	1190 (35.6)		
Academic/research program	15 015 (33.3)	1106 (33.1)		
Integrated network cancer program	9018 (20.0)	663 (19.9)		
Unknown	2337 (5.2)	160 (4.8)		5.2
Clinical stage, *n* (%)				
Stage II	14 482 (32.1)	1311 (39.3)	**< 0.001**	
Stage III	30 630 (67.9)	2029 (60.8)		
Upstaged, *n* (%)	1432 (10.3)	266 (20.5)	**< 0.001**	68.5
Histology, *n* (%)				
Adenocarcinoma, NOS	43 431(96.3)	3159 (94.6)	**< 0.001**	
Mucinous adenocarcinoma	1437 (3.2)	151 (4.5)		
Signet ring cell carcinoma	244 (0.5)	30 (0.9)		
Surgical margin status, *n* (%)			**< 0.001**	
No residual tumor	41 456 (91.9)	2407 (72.1)		
Residual tumor	2849 (6.3)	492 (14.7)		
Unknown	807 (1.8)	441 (13.2)		2.6
Lymphovascular invasion, *n* (%)	5082 (11.3)	580 (17.4)	**< 0.001**	
Unknown	14 982 (33.2)	1122 (33.6)		33.2
Perineural invasion, *n* (%)	3604 (8.0)	282 (8.4)	**< 0.001**	48.3
Unknown	21 547 (47.8)	1842 (55.2)		

Abbreviations: IQR, interquartile range; NOS, not otherwise specified.

^a^ Available data from 2020 cut‐offs were used. Bold *p*‐values are significant at a 0.05 level.

On multivariate analysis, factors independently associated with receipt of adjuvant RT treatment included pathologic upstaging (adjusted odds ratio [aOR] = 1.63, 95% confidence interval [CI]: 1.31–2.02), positive surgical margins (aOR = 1.78, 95% CI: 1.43–2.22), and lymphovascular invasion (aOR = 1.96, 95% CI: 1.66–2.31). Stage III disease (aOR = 0.74, 95% CI: 0.63–0.88) and lack of insurance (aOR = 0.61, 95% CI: 0.38–0.99) were associated with lower odds of receiving adjuvant RT (Table 2 and Figure 2). All associations were consistent in the MICE sensitivity analysis (Appendix A).

**Table 2:** Univariate and multivariate analysis of factors associated with receipt of adjuvant RT.

	Univariate logistic regression			Multivariate logistic regression		
	Unadjusted odds ratio	95% Confidence interval	*p*‐value	Adjusted odds ratio	95% Confidence interval	*p*‐value
Participant characteristics						
Sex						
Male	ref			—	—	—
Female	1.01	1.00–1.01	**< 0.001**	1.06	0.91–1.23	0.442
Race						
White	ref			ref		
Black	1.19	1.05–1.35	**0.005**	1.23	0.96–1.58	0.107
Others	0.94	0.82–1.07	0.348	0.91	0.68–1.23	0.556
Insurance						
Government	ref			ref		
Private	0.89	0.83–0.96	**0.002**	0.90	0.77–1.04	0.160
None	0.71	0.57–0.89	**0.003**	0.61	0.38–0.99	**0.044**
Charlson‐Deyo Score						
0	ref			ref		
1	1.06	0.96–1.17	0.258	1.02	—	—
2	1.01	0.84–1.21	0.942	0.96	—	—
3	1.14	0.92–1.39	0.228	1.07	—	—
Median income quartiles^a^						
< $46 277	ref			ref		
$46 277–$57 856	0.84	0.74–0.95	**0.004**	0.86	0.68–1.09	0.223
$57 857–$74 062	0.91	0.81–1.02	0.122	0.94	0.73–1.19	0.588
$74 063+	0.83	0.74–0.93	**0.001**	0.88	0.67–1.15	0.351
City/Facility characteristics						
Population density						
Rural	ref			ref		
Urban	0.95	0.75–1.20	0.663	0.86	—	—
Metro	1.07	0.85–1.34	0.566	0.91	—	—
Percent no high school degree^a^						
> 15.3%	ref			ref		
9.1%–15.2%	0.93	0.84–1.03	0.172	0.98	0.87–1.11	0.764
5.0%–9.0%	0.90	0.81–1.00	**0.043**	0.95	0.83–1.08	0.416
< 5%	0.85	0.75–0.95	**0.005**	0.92	0.78–1.09	0.356
Type of facility						
Community cancer program	ref			ref		
Comprehensive community cancer program	1.04	0.77–1.42	0.778	—	—	—
Academic/research program	0.88	0.64–1.21	0.431	—	—	—
Integrated network cancer program	0.97	0.70–1.35	0.854	—	—	—
Clinical stage						
Stage II	ref			ref		
Stage III	0.73	0.68–0.79	**< 0.001**	0.74	0.63–0.88	**< 0.001**
Upstaged	2.26	1.95–2.61	**< 0.001**	1.63	1.31–2.02	**< 0.001**
Histology						
Adenocarcinoma	ref			ref		
Mucinous adenocarcinoma	1.44	1.22–1.71	**< 0.001**	1.05	0.77–1.44	0.764
Signet‐cell carcinoma	1.69	1.15–2.47	**0.007**	0.82	0.34–1.94	0.644
Surgical margin status						
No residual tumor	ref			ref		
Residual tumor	2.97	2.68–3.30	**< 0.001**	1.78	1.43–2.22	**< 0.001**
Lymphovascular invasion	1.75	1.58–1.93	**< 0.001**	1.96	1.66–2.31	**< 0.001**
Perineural invasion^a^	1.28	1.12–1.47	**< 0.001**	—	—	—

^a^ Excluded from multivariate analysis due to missingness in data.

**Figure 2:**
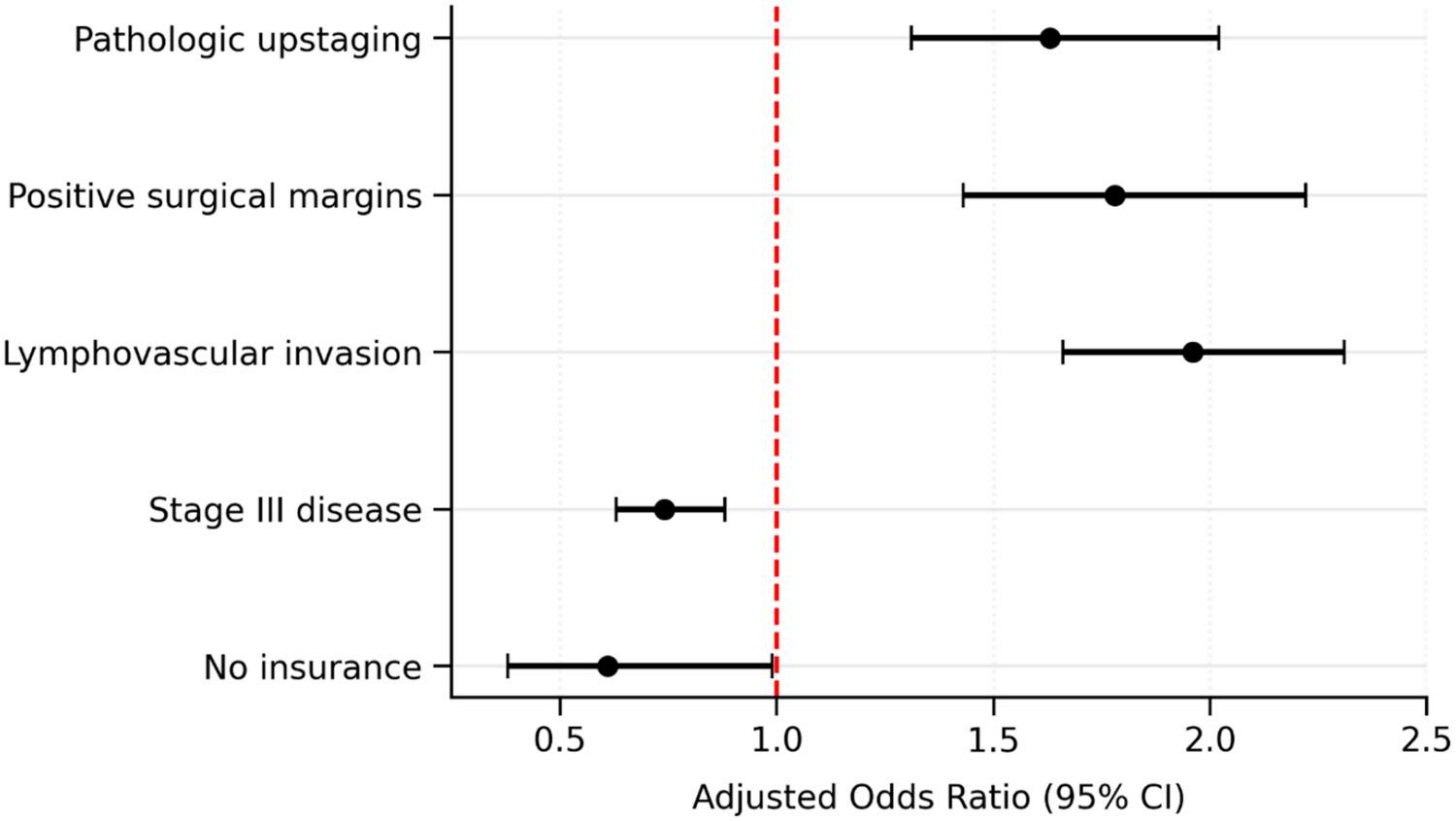
Forest plot illustrating the multivariable logistic regression analysis for factors associated with adjuvant radiotherapy (RT) for locally advanced rectal cancer.

The proportion of patients receiving neoadjuvant RT was stable around 90%, from 2015 to 2022, with only minimal year‐to‐year variation. In addition, the use of adjuvant RT remained at approximately 10% and showed little change over the study period (Figure 3).

**Figure 3:**
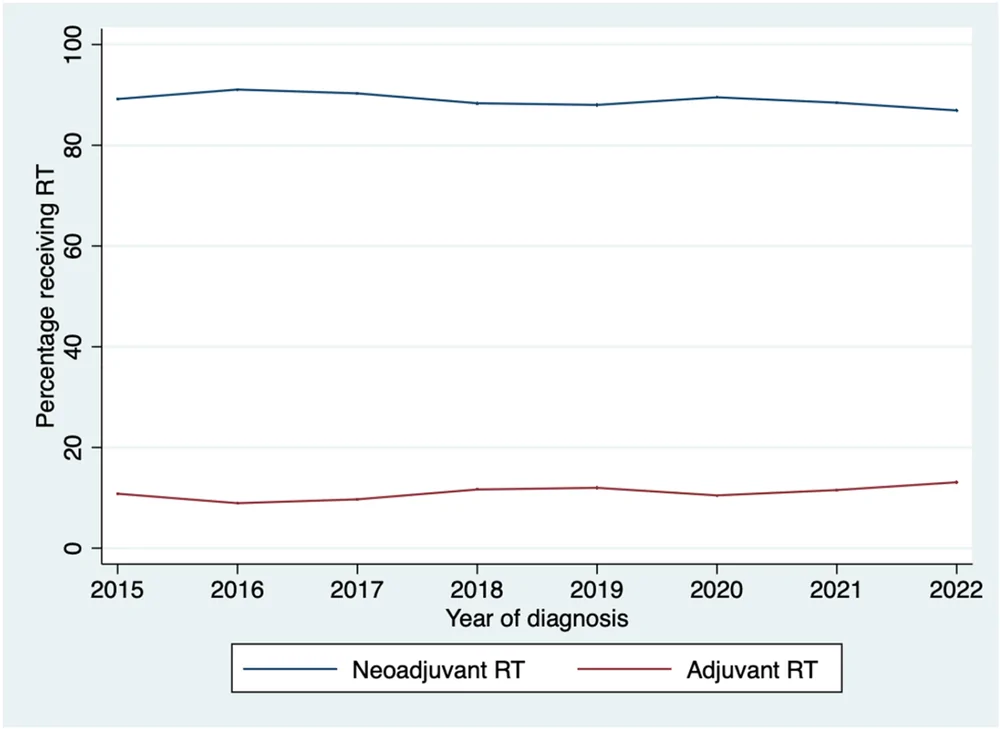
Trends in neoadjuvant and adjuvant radiotherapy (RT) between 2015 and 2022.

## 4 Discussion

The results of this study demonstrate that adverse tumor characteristics—including pathologic upstaging, lymphovascular invasion, and positive surgical margins—were associated with the receipt of adjuvant RT. Therefore, in this cohort of patients with locally advanced disease, the association between aggressive pathology and adjuvant RT suggests that postoperative RT was used to compensate for the omission of a neoadjuvant approach.

In patients initially diagnosed with early‐stage rectal cancer who are subsequently upstaged or present with high‐risk features—such as close (≤ 1 mm) circumferential resection margin, T4 disease, intraoperative perforation, or N1c/N2 nodal involvement—postoperative RT may be selectively considered [13]. While omission of neoadjuvant therapy may be appropriate in such cases, our study population consisted of patients diagnosed with LARC on presurgical evaluation, for whom neoadjuvant RT delivered as part of total neoadjuvant therapy—all chemoradiation administered before surgery—represents the guideline‐recommended standard, regardless of tumor pathology. Although total neoadjuvant therapy was introduced in 2016 and endorsed as the preferred approach in 2018, preoperative RT has remained the standard of care for LARC since the early 2000s [14, 15]. Accordingly, the use of adjuvant RT in these cases reflects variation from guideline‐recommended RT sequencing.

We anticipated the role of sociodemographic factors such as race, education, and residential setting (rural vs. urban) in receipt of adjuvant RT however, no associations were observed on multivariate analysis. A previous study found that patients of racial/ethnic minorities (Blacks, Hispanics, Asians) and low socioeconomic status were less likely to receive the recommended treatments for colorectal cancer compared to White people [16]. In another study, approximately 10% of patients with rectal cancer did not receive guideline‐recommended treatment; among these, the vast majority (> 90%) were treated with adjuvant chemoradiation [17]. Factors associated with receipt of nonstandard care included older age, Black race, and treatment at community cancer programs.

As with any large database study, this analysis is subject to limitations in data quality, accuracy, and missingness. Socioeconomic variables such as income and education are measured at the zip code level and therefore serve only as proxies rather than individual‐level measures. Missingness in certain variables required multiple imputation, which may introduce bias despite best efforts. In addition, the NCDB does not capture the clinical decision‐making process, therefore, it is not possible to determine the rationale underlying treatment sequencing. Adjuvant RT may have reflected planned treatment strategies, patient‐specific considerations, barriers to preoperative therapy, contraindications to neoadjuvant RT, urgent presentations requiring upfront surgery (e.g., obstruction or perforation), or other factors not captured in the database. Therefore, we hesitate to characterize these findings as definitively guideline‐discordant care. Moreover, because patients who did not receive RT were excluded from this study, we cannot determine whether RT would have been administered in the absence of the high‐risk pathological features that appear to be associated with adjuvant RT use.

## 5 Conclusions

Despite longstanding evidence supporting preoperative RT for clinically staged LARC, a small proportion of patients in this national cohort received adjuvant RT. Patients receiving adjuvant RT were more likely to have adverse pathological features, suggesting that postoperative RT may have been used in response to high‐risk findings identified after surgery.

## Author Contributions

A.K. contributed to the conceptualization, investigation, data curation, investigation, methodology, and drafting and editing of the manuscript. M.S., D.A., H.L., and A.T.H. contributed to conceptualization, data curation, investigation, and review and editing of the manuscript.

## Funding

The authors have nothing to report.

## Disclosures

The authors have nothing to report.

## Synopsis

This study aims to evaluate factors associated with the use of adjuvant radiotherapy in the treatment of locally advanced rectal cancer in contemporary practice. Our results show that adverse tumor features were associated with receipt of adjuvant RT. In patients with locally advanced disease, this pattern suggests that postoperative RT may be used to offset the absence of neoadjuvant therapy.

## Supporting information


Supporting File

## Data Availability

The data that support the findings of this study are available from the National Cancer Database (NCDB). Restrictions apply to the availability of these data, which were used under license for this study.

## Appendix A

### A.1

Table A1. Univariate and multivariate analysis of factors associated with receipt of adjuvant RT after multiple imputations.

**Table:**

	Univariate logistic regression			Multivariate logistic regression		
	Unadjusted odds ratio	95% Confidence interval	*p*‐value	Adjusted odds ratio	95% Confidence interval	*p*‐value
Participant characteristics						
Sex						
Male	ref			—	—	—
Female	1.04	0.96–1.11	0.326	—	—	—
Race						
White	ref			ref		
Black	1.19	1.05–1.35	**0.005**	1.12	0.97–1.28	0.116
Others	0.94	0.82–1.07	0.348	0.95	0.82–1.11	0.536
Insurance						
Government	ref			ref		
Private	0.89	0.83–0.96	**0.002**	0.98	0.90–1.06	0.580
None	0.71	0.57–0.89	**0.003**	0.75	0.58–0.95	**0.017**
Charlson‐Deyo Score						
0	ref			ref		
1	1.06	0.96–1.17	0.258	1.02	—	—
2	1.01	0.84–1.21	0.942	0.96	—	—
3	1.14	0.92–1.39	0.228	1.07	—	—
Median income ouartiles^a^						
< $46 277	ref			ref		
$46 277–$57 856	0.86	0.76–0.97	**0.018**	0.92	0.81–1.06	0.251
$57 857–$74 062	0.92	0.82–1.04	0.189	0.99	0.86–1.15	0.938
$74 063+	0.85	0.76–0.95	**0.005**	0.97	0.83–1.13	0.680
City/Facility characteristics						
Population density						
Rural	ref			ref		
Urban	0.95	0.75–1.20	0.663	0.86	—	—
Metro	1.07	0.85–1.34	0.566	0.91	—	—
Percent no high school degree^a^						
> 15.3%	ref			ref		
9.1%–15.2%	0.94	0.84–1.05	0.250	0.98	0.87–1.11	0.764
5.0%–9.0%	0.91	0.82–1.01	0.081	0.95	0.83–1.08	0.416
< 5%	0.87	0.77–0.98	**0.026**	0.92	0.78–1.09	0.356
Type of facility						
Community cancer program	ref			ref		
Comprehensive community cancer program	0.91	0.78–1.05	0.204	—	—	—
Academic/Research program	0.90	0.78–1.05	0.175	—	—	—
Integrated network cancer program	0.90	0.77–1.05	0.190	—	—	—
Clinical stage						
Stage II	ref			ref		
Stage III	0.73	0.68–0.79	**< 0.001**	0.74	0.68–0.80	**< 0.001**
Upstaged	1.44	1.24–1.66	**< 0.001**	1.27	1.09–1.48	**0.003**
Histology						
Adenocarcinoma	ref			ref		
Mucinous adenocarcinoma	1.441	1.22–1.71	**< 0.001**	1.35	1.12–1.62	**0.001**
Signet‐cell carcinoma	1.69	1.15–2.47	**0.007**	1.17	0.76–1.81	0.464
Surgical margin status						
No residual tumor	ref			ref		
Residual tumor	2.97	2.68–3.30	**< 0.001**	2.76	2.48–3.08	**< 0.001**
Lymphovascular invasion	1.47	1.33–1.63	**< 0.001**	1.39	1.25–1.56	**< 0.001**
Perineural invasion	1.21	1.09–1.35	**0.001**	1.00	0.87–1.14	0.971

a Available data from 2020 cut‐offs were used. Bold *p*‐values are significant at a 0.05 level.
